# Invasive and antiplatelet treatment of patients with non‐ST‐segment elevation myocardial infarction: Understanding and addressing the global risk‐treatment paradox

**DOI:** 10.1002/clc.23232

**Published:** 2019-07-17

**Authors:** Ingo Ahrens, Oleg Averkov, Eduardo C. Zúñiga, Alan Y. Y. Fong, Khalid F. Alhabib, Sigrun Halvorsen, Muhamad A. B. S. K. Abdul Kader, Ricardo Sanz‐Ruiz, Robert Welsh, Hongbin Yan, Philip Aylward

**Affiliations:** ^1^ Augustinerinnen Hospital, Academic Teaching Hospital University of Cologne Cologne Germany; ^2^ Pirogov Russian National Research Medical University Moscow Russia; ^3^ Clinica de Occidente Santiago de Cali Colombia; ^4^ Department of Cardiology Sarawak Heart Centre Kota Samarahan Malaysia; ^5^ Department of Cardiac Sciences, King Fahad Cardiac Centre College of Medicine, King Saud University Riyadh Saudi Arabia; ^6^ Oslo University Hospital Ullevål Norway; ^7^ Penang Hospital Pulau Pinang Malaysia; ^8^ Gregorio Marañón Hospital and Complutense University Madrid Spain; ^9^ Mazankowski Alberta Heart Institute and University of Alberta Edmonton Alberta Canada; ^10^ Fuwai Hospital Beijing China; ^11^ South Australian Health and Medical Research Institute Flinders University and Medical Centre Adelaide Australia

**Keywords:** antiplatelet therapy, early invasive strategy, non‐ST‐segment elevation myocardial infarction, treatment‐risk paradox

## Abstract

Clinical guidelines for the treatment of patients with non‐ST‐segment elevation myocardial infarction (NSTEMI) recommend an invasive strategy with cardiac catheterization, revascularization when clinically appropriate, and initiation of dual antiplatelet therapy regardless of whether the patient receives revascularization. However, although patients with NSTEMI have a higher long‐term mortality risk than patients with ST‐segment elevation myocardial infarction (STEMI), they are often treated less aggressively; with those who have the highest ischemic risk often receiving the least aggressive treatment (the “treatment‐risk paradox”). Here, using evidence gathered from across the world, we examine some reasons behind the suboptimal treatment of patients with NSTEMI, and recommend approaches to address this issue in order to improve the standard of healthcare for this group of patients. The challenges for the treatment of patients with NSTEMI can be categorized into four “P” factors that contribute to poor clinical outcomes: *p*atient characteristics being heterogeneous; *p*hysicians underestimating the high ischemic risk compared with bleeding risk; *p*rocedure availability; and *p*olicy within the healthcare system. To address these challenges, potential approaches include: developing guidelines and protocols that incorporate rigorous definitions of NSTEMI; risk assessment and integrated quality assessment measures; providing education to physicians on the management of long‐term cardiovascular risk in patients with NSTEMI; and making stents and antiplatelet therapies more accessible to patients.

## 1 INTRODUCTION

### 1.1 Disease burden of non‐ST‐segment elevation myocardial infarction

Non‐ST‐segment elevation myocardial infarction (NSTEMI) is the leading cause of emergency hospitalization for acute coronary syndrome (ACS) in Europe and North America.^1^, ^2^, ^3^, ^4^ Although both patients with NSTEMI and ST‐segment elevation myocardial infarction (STEMI) are at a high risk of recurrent cardiovascular events, patients with NSTEMI have higher long‐term mortality and cardiovascular risk than those with STEMI.^5^, ^6^, ^7^, ^8^ Furthermore, the proportion of patients with acute myocardial infarction (MI) who have NSTEMI is increasing relative to those with STEMI.^9^, ^10^, ^11^

Results from the French Registry of Acute ST‐Elevation or Non‐ST‐Elevation Myocardial Infarction (FAST‐MI) and the Swedish Web‐system for Enhancement and Development of Evidence‐based care in Heart disease Evaluated According to Recommended Therapies (SWEDEHEART) registry showed that the 6‐month and 1‐year mortality of patients with STEMI or NSTEMI have generally decreased since 1995.^11^, ^12^ However, since 2010, there has been no improvement in the 6‐month mortality of patients with NSTEMI, regardless of whether or not they received percutaneous coronary intervention (PCI); in striking contrast, mortality has continued to decline in patients with STEMI during this time.^11^

### 1.2 Guideline recommendations for invasive and antiplatelet treatment of patients with NSTEMI

The standard of care for patients with NSTEMI at high ischemic risk is an early invasive strategy with cardiac catheterization within 24 hours of the onset of symptoms, with prompt revascularization using PCI or coronary artery bypass graft (CABG) surgery as clinically indicated.^13^, ^14^, ^15^ Major international guidelines also recommend initiation of at least 12 months of dual antiplatelet therapy (DAPT) with aspirin and a P2Y_12_ inhibitor (ticagrelor, prasugrel, or clopidogrel) in patients with NSTEMI who are managed with medical therapy and/or who are treated with revascularization (ie, irrespective of initial treatment strategy), unless there are previous or ongoing contraindications.^13^, ^14^, ^15^, ^16^, ^17^, ^18^ Ticagrelor is recommended over clopidogrel for patients with NSTEMI, including those pretreated with clopidogrel (which should be discontinued when ticagrelor is started). Ticagrelor is contraindicated in patients with previous intracranial hemorrhage or ongoing bleeds.^13^, ^14^, ^15^, ^16^, ^17^, ^18^ Prasugrel is recommended for patients with NSTEMI who have received angiography and are undergoing PCI. However, prasugrel is not recommended for patients in whom coronary anatomy is unknown and an indication for PCI is not clearly established; patients who are 75 years of age or older; or patients with a body weight of less than 60 kg. Prasugrel is contraindicated in patients with previous intracranial hemorrhage, previous ischemic stroke or transient ischemic attack, or ongoing bleeds.^13^, ^14^, ^15^, ^16^, ^17^, ^18^ Clopidogrel is a less potent P2Y_12_ inhibitor than ticagrelor and prasugrel; it is recommended for patients who cannot receive ticagrelor or prasugrel, or who require oral anticoagulation.^13^, ^14^, ^15^, ^16^, ^17^, ^18^

Clinical risk scores, such as the DAPT score, can help guide decisions around whether to continue antiplatelet therapy beyond 1 year, by providing a risk‐benefit ratio based on age, smoking status, comorbidity, and medical history.^19^ For patients with NSTEMI who are elderly and have a high comorbidity burden, conservative treatment could be a reasonable approach.^16^, ^19^

### 1.3 Invasive strategy and P2Y_12_ inhibitor treatment evidence for NSTEMI

An early invasive strategy of cardiac catheterization has a central role in the management of patients with NSTEMI because it facilitates: confirmation of the diagnosis of ACS related to obstructive epicardial coronary artery disease; identification of the culprit lesion(s); the establishment of the indication for revascularization using PCI or CABG; the stratification of the patient's short‐ and long‐term ischemic risk.^15^ Compared with a more conservative strategy, an early invasive strategy has been shown to improve clinical outcomes and reduce recurrent ACS episodes, subsequent rehospitalization, and revascularization.^15^ A meta‐analysis of seven randomized clinical trials including 8375 patients with NSTEMI showed that, vs. a conservative strategy, an early invasive strategy was associated with lower incidences of 2‐year all‐cause mortality (4.9% vs. 6.5%; risk ratio [RR]: 0.75; 95% confidence interval [CI]: 0.63‐0.90; *P* = .001), 2‐year nonfatal MI (7.6% vs. 9.1%; RR: 0.83; 95% CI: 0.72‐0.96; *P* = .012), and 13‐month rehospitalization for recurrent ACS (19.9% vs. 28.7%; RR: 0.69; 95% CI: 0.65‐0.74; *P* = .0001).^20^ A separate meta‐analysis of individual patient data from 5467 patients across three randomized controlled trials with a 5‐year follow‐up period showed that, vs. a selective invasive strategy, a routine invasive strategy was associated with a lower risk of cardiovascular death or nonfatal MI (14.7% vs. 17.9%; hazard ratio [HR]: 0.81; 95% CI: 0.71‐0.93; *P* = .002) and lower risk of MI (10.0% vs. 12.9%; HR: 0.77; 95% CI: 0.65‐0.90; *P* = .001).^21^ There was an absolute risk reduction of 2.0%, 3.8%, and 11.1% in low‐, intermediate‐, and high‐risk patients receiving a routine invasive strategy, respectively.^21^ Registry data from six Arabian Gulf countries showed that patients with NSTEMI managed with PCI and CABG had better outcomes than those treated conservatively.^22^

Guideline‐indicated treatment of patients with NSTEMI with potent P2Y_12_ inhibitors is mainly based on evidence from the Trial to Assess Improvement in Therapeutic Outcomes by Optimizing Platelet InhibitioN with Prasugrel‐Thrombolysis in Myocardial Infarction (TRITON‐TIMI 38) and the PLATelet Inhibition and Patient Outcomes (PLATO) study. TRITON‐TIMI 38 enrolled patients scheduled for PCI. PLATO enrolled both invasively and noninvasively managed patients. In TRITON‐TIMI 38, prasugrel showed a reduction in the primary composite efficacy endpoint of cardiovascular death, MI, or stroke vs. clopidogrel in the NSTEMI patient subgroup (HR: 0.85; 95% CI: 0.73‐0.97).^23^, ^24^ Prasugrel was associated with increased non‐CABG major bleeding compared with clopidogrel (HR: 1.40; 95% CI: 1.05‐1.88). The PLATO study showed a reduction in the primary efficacy endpoint of cardiovascular death, MI, or stroke for ticagrelor vs. clopidogrel for the NSTEMI patient subgroup (HR: 0.83; 95% CI: 0.73‐0.94), with no significant increase in major bleeding.^25^, ^26^ Primary efficacy and safety endpoints from the two trials are summarized in Table 1 (note: differences in study design, patient populations, and endpoint assessments mean that cross‐trial comparisons are not appropriate). These clinical trial results are supported by real‐world evidence data from the SWEDEHEART registry, showing the superiority of ticagrelor to clopidogrel for the prevention of cardiovascular events in patients with NSTEMI.^27^

**Table 1:** Primary efficacy and safety endpoints in patients with NSTE‐ACS and NSTEMI in the TRITON‐TIMI 38 and PLATO trials

	Event rate	HR (95% CI)	*P*	ARR^a^	RRR^b^	NNT^c^	NNH^c^
NSTE‐ACS population							
Primary efficacy endpoint^d^							
TRITON	Prasugrel: 9.30% Clopidogrel: 11.23%	0.82 (0.73‐0.93)	0.0015	1.93%	17.2%	52	—
PLATO	Ticagrelor: 10.0% Clopidogrel: 12.3%	0.83 (0.74‐0.93)	0.0013	2.3%	18.7%	43	—
CV death							
TRITON	Prasugrel: 1.78% Clopidogrel: 1.83%	0.98 (0.73‐1.31)	0.8853	0.05%	2.7%	2000	—
PLATO	Ticagrelor: 3.7% Clopidogrel: 4.9%	0.77 (0.64‐0.93)	0.0070	1.2%	24.5%	83	—
MI							
TRITON	Prasugrel: 7.26% Clopidogrel: 9.46%	0.76 (0.66‐0.87)	0.0001	2.20%	23.3%	45	—
PLATO	Ticagrelor: 6.6% Clopidogrel:7.7%	0.86 (0.74‐0.99)	0.0419	1.1%	14.3%	91	—
Stroke							
TRITON	Prasugrel: 0.97% Clopidogrel: 0.91%	1.07 (0.71‐1.60)	0.7481	−0.06%	−6.6%	—	1667
PLATO	Ticagrelor: 1.3% Clopidogrel: 1.4%	0.95 (0.69‐1.33)	0.79	0.1%	7.1%	1000	—
Primary safety endpoint^e^							
TRITON	Prasugrel: 2.16% Clopidogrel: 1.55%	1.40 (1.05‐1.88)	0.0223	−0.61%	−39.4%	—	164
PLATO	Ticagrelor: 13.4% Clopidogrel: 12.6%	1.07 (0.95‐1.19)	0.26	−0.8%	−6.3%	—	125
NSTEMI population							
Primary efficacy endpoint^d^							
TRITON	Prasugrel: 9.5% Clopidogrel: 11.2%	0.85 (0.73‐0.97)	0.019	1.7%	15.2%	59	—
PLATO	Ticagrelor: 11.4% Clopidogrel: 13.9%	0.83 (0.73‐0.94)	NR	2.5%	18.0%	40	—
Primary safety endpoint^e^							
TRITON	Prasugrel: 2.0% Clopidogrel: 1.5%	1.38 (0.97‐1.96)	0.019	−0.5%	−33.3%	—	200
PLATO	Ticagrelor: 14.7% Clopidogrel: 14.3%	1.02 (0.90‐1.15)	NR	−0.4%	−2.8%	—	250

*Note*: Differences in study design, patient populations and endpoint assessments make cross‐trial comparisons inappropriate.

Abbreviations: AR, absolute risk; ARR, absolute risk reduction; CV, cardiovascular; HR, hazard ratio; MI, myocardial infarction; NNH, number needed to harm; NNT, number needed to treat; NR, not reported; NSTE‐ACS, non‐ST‐segment elevation acute coronary syndrome; NSTEMI, non‐ST‐segment elevation acute coronary syndrome; myocardial infarction; RRR, relative risk reduction.

a TRITON: event rate in clopidogrel group minus event rate in prasugrel group; PLATO: event rate in clopidogrel group minus event rate in ticagrelor group.

b ARR divided by event rate in clopidogrel group.

c 1 divided by ARR. TRITON: per 450 days; PLATO: per 360 days.

d CV death, MI, stroke.

e TRITON: non‐CABG related TIMI major bleeding; PLATO: major bleeding study criteria were bleeding leading to clinically significant disability, or bleeding either associated with a drop in the hemoglobin level of 3 to 5 g/dL or requiring transfusion of 2 to 3 units of red cells.

### 1.4 Suboptimal treatment of patients with NSTEMI

Despite the above recommendations and findings, patients with NSTEMI often receive less aggressive secondary prevention treatment than patients with STEMI.^8^, ^28^ Moreover, patients who have the highest ischemic risk often receive the least aggressive treatment, including both invasive and medical management; a phenomenon that has been termed the “treatment‐risk paradox.”^29^, ^30^, ^31^, ^32^

Suboptimal treatment of patients with multiple ischemic risk factors was highlighted in the Pattern of Repeat Cardiovascular Events During Follow‐up After First Diagnosis Event‐MI‐2 (PRECLUDE‐2) registry study.^33^ Ischemic risk factors consisted of multivessel disease, diabetes mellitus, chronic kidney disease, prior MI, and age of at least 65 years.^33^ Results from the PRECLUDE‐2 study, which included invasively managed patients with MI, showed a higher ischemic risk compared with bleeding risk during a median follow‐up of 3.6 years; having five ischemic risk factors, compared with only one risk factor, was associated with a 5 to 9 times increased incidence of ischemic events and a 2 to 4 times increased incidence of major bleeding.^33^ The incidence of ischemic events increased with increasing number of ischemic risk factors, highlighting an unmet need for additional preventive measures in these high‐risk patients.^33^

The challenges for the treatment of patients with NSTEMI can be categorized into four “P” factors that contribute to poor clinical outcomes in these patients: *p*atient characteristics, *p*hysician guideline implementation, *p*rocedure availability, and *p*olicy within the healthcare system. Here, we will consider each in turn and suggest some solutions to address these challenges in order to improve the standard of healthcare for this group of patients.

## 2 CHALLENGES FOR THE TREATMENT OF PATIENTS WITH NSTEMI

### 2.1 Patient characteristics

Patients with NSTEMI present with more heterogeneous characteristics than patients with STEMI, with a wide variation in ischemic risk and comorbid conditions, making NSTEMI more challenging to diagnose and treat in these patients.^34^ Whereas patients with STEMI typically have complete occlusion of a large epicardial coronary artery, patients with NSTEMI are often affected by multiple variable factors; including varying degrees of reduction of coronary flow, atherosclerotic changes in the vessel wall, calcification, plaque rupture, and subsequent nonocclusive intracoronary thrombus formation.^35^ Indeed, in a study of patients hospitalized with MI who underwent coronary angiography in Alberta, Canada, of the 2092 patients with MI with nonobstructive coronary arteries, 1542 (73.7%) received a diagnosis of NSTEMI, and 550 (26.3%) a diagnosis of STEMI.^36^

There is also heterogeneity in the demographics of NSTEMI patient populations across the world, as indicated by the variety of patient demographic data across different countries.^37^, ^38^, ^39^ This heterogeneity could partly explain the variation in mortality of patients with cardiovascular disease between countries.^37^, ^38^, ^40^

Furthermore, patients with NSTEMI are more likely to be older in age and have a higher rate of comorbidities, such as diabetes, impaired renal function, and lung disease, than patients with STEMI.^41^, ^42^ These comorbidities contribute to a greater burden of coronary artery disease and an increased risk of cardiovascular events for patients with NSTEMI, and therefore lead to increased long‐term mortality.^5^, ^15^

**Figure 1:**
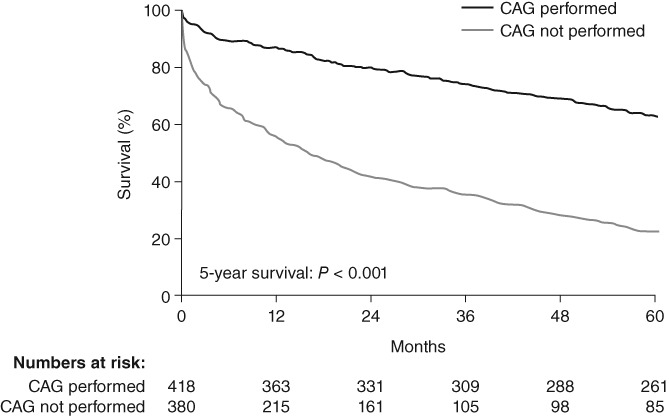
Kaplan‐Meier survival curves showing all‐cause mortality rates for patients with NSTEMI who did not undergo coronary angiography vs. those who did. Adapted from Feldman et al. with permission from SAGE Ltd.^43^ Abbreviation: CAG, coronary angiography

The wide variation in risk in patients with NSTEMI affects treatment decisions. Some patients are considered at too low risk of recurrent cardiovascular events to warrant an invasive strategy, whereas others are regarded as “too sick” to undergo coronary angiography and/or subsequent revascularization because of advanced age or severe comorbidities.^43^ Figure 1 shows the benefit of invasive management vs. medical management on the survival of patients with NSTEMI.

Diagnosis of NSTEMI is also less straightforward than that of STEMI, which can be identified rapidly based on an electrocardiogram (ECG) measurement. The identification of patients with NSTEMI is often delayed owing to the frequent lack of definitive ECG changes and uncertainty about the definition of NSTEMI with regard to elevated cardiac troponin levels.^42^ Implementation of high‐sensitivity cardiac troponin assays will lead to an increase in the diagnosis of NSTEMI.^44^ Age, sex, comorbidities, and in‐hospital management strategies (eg, PCI or medical management) may also influence decisions in patients with NSTEMI regarding prescription at discharge.^28^, ^45^

### 2.2 Physician guideline implementation

Another challenge for the treatment of patients with NSTEMI is the underestimation of the high ischemic risk compared with bleeding risk in these patients, which contributes to the suboptimal use of treatments and is suggestive of barriers to guideline implementation.^31^, ^41^

In the ACS II Canadian registry, the most common reason for not choosing an invasive treatment strategy in patients with NSTEMI was an underestimation of ischemic risk by physicians, even though a large proportion of these patients were at intermediate to high risk according to their Thrombolysis in Myocardial Infarction (TIMI) risk score.^46^, ^47^ Results from the registry also showed weak correlations between risk assessment by physicians and TIMI and Global Registry of Acute Coronary Events (GRACE) risk scores,^48^ which are recommended for guiding treatment decisions for patients with NSTEMI in international guidelines.^13^, ^15^

Objective risk assessment using the GRACE risk score provided superior risk discrimination to physician‐perceived risk for 6‐month mortality in patients with ACS in the Perceived Risk of Ischemic and Bleeding Events in Acute Coronary Syndrome Patients (PREDICT) study.^49^ Here, physicians were shown to overestimate the risk of 6‐month mortality among patients with a low GRACE score and underestimate risk among those with a high GRACE score, consistent with the treatment‐risk paradox.^49^

Patients with NSTEMI at high ischemic risk were also not treated optimally in the Myocardial Ischaemia National Audit Project (MINAP) registry, in which the use of guideline‐indicated care for patients with NSTEMI decreased with increasing GRACE risk score, even though optimal guideline‐indicated care was associated with greater survival gains for high‐risk patients.^31^

Adherence to guideline recommendations for the management of patients with NSTEMI varies widely, as demonstrated in a systematic review of 45 studies conducted around the world; showing adherence rates varying within and across studies from approximately 5.0% to 95.0% for recommended pharmacological treatment, and from 16.0% to 95.8% for coronary angiography.^50^ Factors related to lower adherence to guideline recommendations included patients being of older age, female gender, presence of comorbidities, not having a cardiologist as their primary care provider and being treated in a hospital with no PCI/CABG facilities; having no health insurance was related to lower rates of coronary angiography but not medication prescription rates. A large proportion of patients with NSTEMI may therefore not be receiving guideline‐indicated care, which may have prognostic implications.^50^ Indeed, a cohort study using data from MINAP showed that of patients with NSTEMI eligible to receive care, 337 881 (86.9%) missed receiving at least one or more guideline‐indicated intervention; of whom 66.3% and 43.4% did not receive P2Y_12_ inhibitors and coronary angiography, respectively.^51^ Accelerated failure time models were used to quantify the impact of nonadherence on survival. They showed that if all eligible patients in this study had received optimal care in accordance with guidelines available during the study period, then 32 765 (28.9%) deaths (95% CI: 30 531‐33 509) may have been prevented.^51^ Furthermore, data from national registries have indicated that patients with NSTEMI are more likely to receive clopidogrel than the potent P2Y_12_ inhibitors,^29^, ^52^, ^53^ which are generally preferentially recommended in international guidelines.^13^, ^15^, ^16^, ^17^

There is also evidence to suggest that patients with NSTEMI may be less likely to be treated in academic medical centers than patients with STEMI, and therefore less likely to be directed to larger hospitals with catheterization laboratories; indicating some degree of referral bias toward patients with STEMI.^42^

The risk may be underestimated by physicians based on the intensity of treatment the patients are receiving and the advanced age of the patient, despite evidence that an early intensive strategy in the eldest patients with NSTEMI is associated with the greatest reduction in 1‐year mortality.^47^, ^48^, ^49^, ^54^ Physicians' and/or patients' concerns about the risk of complications with an invasive strategy may also affect the implementation of guideline‐indicated treatment. Furthermore, physicians may attribute mortality risk to comorbidities rather than the MI itself, even though evidence suggests that patients with NSTEMI have an increased risk of death beyond what can be explained by comorbidities.^41^ Together, this evidence suggests that guidelines on risk stratification in patients with NSTEMI are insufficiently implemented, which may partly explain why the treatment of patients with NSTEMI is suboptimal.^28^

### 2.3 Procedure availability and policy within the healthcare system

Optimal guideline‐indicated treatment of patients with NSTEMI is also affected by the availability of procedures, as well as governmental and hospital policies. Several studies have demonstrated wide geographical variation in the use of guideline‐indicated treatments for patients with NSTEMI, which is linked to hospital and geographical characteristics.^30^, ^55^, ^56^, ^57^

In the SNAPSHOT ACS study, assessing patients with chest pain admitted to hospitals in Australia and New Zealand, the proportion of patients with NSTEMI who were given a coronary angiogram varied from 56.5% to 68.9% across health jurisdictions, whereas the proportion of patients with STEMI/left bundle branch block given a coronary angiogram ranged from 81.1% to 100%.^30^

Registry data from Arabian Gulf countries (Saudi Arabia, Bahrain, Qatar, Oman, United Arab Emirates, and Yemen) showed that only 26.8% of patients with NSTEMI received coronary angiography and 21.9% received revascularization with either PCI or CABG.^22^ The rate of conservative management varied according to the country, and the relatively low rate of invasive management may be explained by the fact that fewer than half of the hospitals in the registry had an on‐site catheterization laboratory.^22^ Access to hospitals with catheterization was associated with reduced recurrent adverse cardiovascular events in patients with ACS.^58^

A study using the Malaysian National Cardiovascular Disease Database—ACS (NCVD‐ACS) registry showed geographical variations in prescribing rates for secondary preventive medications in patients with NSTEMI.^57^ For example, patients in East Malaysia were less likely to be prescribed P2Y_12_ inhibitors or aspirin than patients in the Western region of the Malaysian Peninsular.^57^

In China, a nationwide database study that included 1055 tertiary hospitals showed an in‐hospital mortality of 3.6% and a PCI utilization rate of 37.2% in patients with NSTEMI (corresponding rates in patients with STEMI were 5.1% and 47.8%, respectively).^55^ The study identified wide variations in the rates of in‐hospital mortality across geographical regions, and the rates were significantly lower in patients who received PCI than in those who did not.^55^ A separate registry study of patients with NSTEMI in China, including 142 hospitals, showed that angiography and PCI were performed in 63.1% and 58.2% of these patients, respectively, and that only 41.7% of patients with the highest risk underwent PCI.^59^

Another study, investigating geographic variation in guideline‐indicated treatments for patients with NSTEMI in the English National Health Service, using data from the MINAP registry, showed that the proportion of patients receiving optimal care was only 13.5%; with P2Y_12_ inhibitor and coronary angiography treatments among the least provided care, and over half (58.1%) of patients not being under the care of a cardiologist.^56^ The provision of both coronary angiography (median, 57.4%; interquartile range [IQR], 48.8%‐66.7%) and P2Y_12_ inhibitors (median, 39.7%; IQR, 32.4%‐46.9%) varied widely across Clinical Commissioning Groups (CCGs), which was explained by differences in the provision of care at the level of the hospital rather than between CCGs.^56^

Geographical variation in care may be due to the lack of availability of cardiologists, catheterization laboratories, and medical treatments in rural areas compared with urban areas.^60^ For example, a study assessing outcomes in patients with acute MI in New South Wales, Australia, showed that patients with NSTEMI presenting to a rural hospital were 70% less likely to undergo cardiac revascularization than patients presenting to an urban hospital.^61^ Geographical variation in care may also be affected by the financial burden of different P2Y_12_ inhibitors to patients worldwide. Prasugrel and ticagrelor are generally more expensive than clopidogrel, which is no longer patented. Decision‐analytical modeling in patients with ACS shows ticagrelor to be cost effective compared with generic clopidogrel across different countries and public healthcare systems, including in Sweden, the United Kingdom, Germany, and Brazil.^62^ Cost‐effectiveness of ticagrelor compared with clopidogrel has also been shown from the China, Singapore, Thailand, and Vietnam healthcare perspectives.^63^, ^64^, ^65^, ^66^

The implications of the mode of patient presentation to hospitals (ie, via emergency medical services [EMS] vs. self‐presentation) were investigated in a study of patients with NSTEMI admitted to a well‐defined metropolitan healthcare region in Edmonton, Alberta, Canada, over 3 months in 2008.^67^ Of the 263 patients included in the study, 78.3% underwent cardiac catheterization, with lower utilization in the EMS group (60.2%) than the self‐presenting group (88.2%; *P* < .001).^67^ There was a significantly lower rate of cardiac catheterization in the patients with a high GRACE risk score (*P* < .001), which was especially apparent in patients who presented by EMS.^67^ Catheterization rates in community hospitals (84.4%) were higher than those in PCI centers (71.9%; *P* = .014) even though patients admitted to PCI centers had an overall higher GRACE risk score.^67^

Aside from differences in the availability of specialist services, variation in healthcare for patients with NSTEMI across hospitals could also be due to differences in: the number of hospital admissions; national guidelines and hospital protocols; clinicians' treatment decisions; cost of and access to new and effective medications; and healthcare system procurement, infrastructure, and funding.^4^, ^34^, ^37^, ^56^, ^57^

## 3 ADDRESSING THE CHALLENGES FOR THE TREATMENT OF PATIENTS WITH NSTEMI

The treatment‐risk paradox in patients with NSTEMI is a global problem that is influenced by the four “P” factors discussed in this paper: *p*atient characteristics, *p*hysician guideline implementation, *p*rocedure availability, and *p*olicy. There are several approaches that can be used to help address these challenges for the treatment of patients with NSTEMI.

It is noteworthy to mention that the definition of non‐ST‐segment elevation ACS refers to both patients with NSTEMI and patients with unstable angina. This definition highlights the heterogeneity of patient characteristics and the wide variation in risk in these patients. For patients with NSTEMI who experience chest pain coupled with elevated serum troponin levels and stenosis, but no ST‐segment elevation on the ECG, a potent P2Y_12_ inhibitor should be the preferred antiplatelet treatment for all patients; except for those at very high bleeding risk or with other contraindications. In contrast, patients with a diagnosis of unstable angina who present with chest pain, without elevated troponin levels or ischemic ECG changes, are a heterogeneous group that requires confirmation of the presence of coronary artery disease with appropriate risk stratification and treatment.

GRACE (https://www.mdcalc.com/grace-acs-risk-mortality-calculator)^68^ and TIMI (https://www.mdcalc.com/timi-risk-score-ua-nstemi)^69^ risk scoring systems have undergone extensive validation and are recommended in international guidelines for guiding treatment decisions for patients with NSTEMI,^13^, ^15^ and should be utilized by physicians (Table 2). Use of the CRUSADE score (http://www.crusadebleedingscore.org/)^70^ may also be considered in patients undergoing coronary angiography to quantify bleeding risk (Table 2).^15^

**Table 2:** Recommended risk scoring systems for the assessment of ischemic and bleeding risk in patients with NSTEMI^15^, ^68^, ^69^, ^70^

	GRACE	TIMI	CRUSADE
Risk measured	Ischemic	Ischemic	Bleeding
Risk estimated	Mortality while in hospital, at 6 months, at 1 year, and at 3 years The combined risk of death or MI at 1 year	Adverse outcome (death, MI, urgent revascularization)	In‐hospital major bleeding event
Variables used to calculate score	Age, systolic blood pressure, pulse rate, serum creatinine, Killip class at presentation, cardiac arrest at admission, elevated cardiac biomarkers, and ST deviation	Age ≥65 years, ≥3 CAD risk factors, known CAD, aspirin use in the past 7 days, severe angina (≥2 episodes within 24 hours), ST change ≥0.5 mm, and positive cardiac marker	Baseline hematocrit, diabetes mellitus, GFR: Cockcroft‐Gault, heart rate on admission, prior vascular disease, sex, signs of CHF on admission, and systolic blood pressure on admission
Online calculator	https://www.mdcalc.com/grace‐acs‐risk‐mortality‐calculator	https://www.mdcalc.com/timi‐risk‐score‐ua‐nstemi	http://www.crusadebleedingscore.org/

Abbreviations: CAD, coronary artery disease; CHF, congestive heart failure; CRUSADE, Can Rapid risk stratification of Unstable angina patients Suppress ADverse outcomes with Early implementation of the ACC/AHA guidelines; GFR, glomerular filtration rate; GRACE, Global Registry of Acute Coronary Events; MI, myocardial infarction; NSTEMI, non‐ST‐segment elevation myocardial infarction; TIMI, Thrombolysis In Myocardial Infarction.

**Figure 2:**
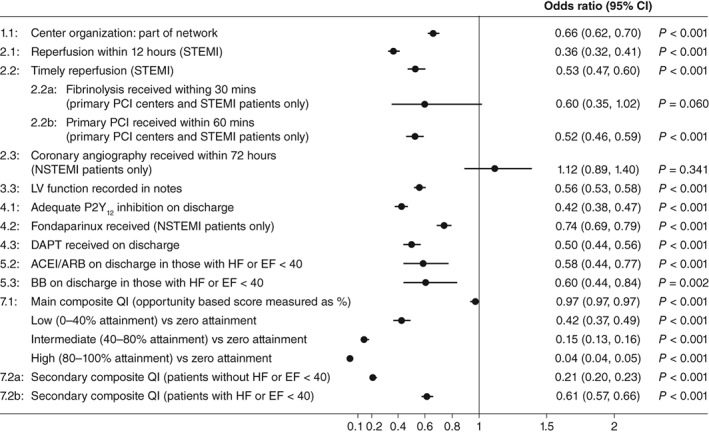
Association between the European Society of Cardiology Acute Cardiovascular Care Association quality indicators for acute myocardial infarction and crude 30‐day mortality. Adapted from Bebb et al.^72^ The composite opportunity QI was divided into the following categories: zero, received no interventions out of those eligible for; low, received <40% of interventions eligible for; intermediate, received ≥40% to <80% of interventions eligible for; and high, received ≥80% of interventions eligible for. Abbreviations: ACEI; angiotensin‐converting enzyme inhibitor; ARB; angiotensin receptor blocker; BB, β‐blocker; CI, confidence interval; DAPT, dual antiplatelet therapy; EF, ejection fraction; HF, heart failure; LV, left ventricular; NSTEMI, non‐ST‐segment elevation myocardial infarction; PCI, percutaneous coronary intervention; QI, quality indicator; STEMI, ST‐segment elevation myocardial infarction

Accurate risk estimation should help address the underestimation of risk in patients with NSTEMI. However, improving guideline implementation and adherence are key to overcoming the problem and optimizing treatment for these patients. We encourage the development of guidelines and hospital protocols that include quality assessment measures for assessing clinical uptake of guideline‐indicated care. Quality measures should encourage optimal evidence‐based treatment. For example, they need to specify which antiplatelet agents should be used rather than only the length of treatment. An example of a quality measure could be that “at least 75% of patients with a GRACE score above 140 must be treated with a potent P2Y_12_ inhibitor, unless contraindicated.” Good reasons need to be provided by physicians for failing to treat patients with a potent P2Y_12_ inhibitor. For example, for patients with a GRACE score of 100 to 130, the use of these treatments might be dependent on the availability of resources. Importantly, the development of guidelines and protocols need to be supported with advocacy for their use, and national registries should be used in order to monitor the progress of improving healthcare for patients with NSTEMI. We recommend consulting the quality indicators recommended by Schiele et al,^71^ which are included in Table 3. In a national cohort study using the MINAP registry, Bebb et al^72^ assessed the performance of hospitals according to the European Society of Cardiology Acute Cardiovascular Care Association quality indicators and 30‐day mortality for acute MI. Eleven quality indicators had a significant inverse association with 30‐day mortality (all *P* < .001), suggesting that quality indicators have the potential to improve patient healthcare and reduce varied mortality from acute MI (Figure 2).

**Table 3:** Summary of the quality indicators: definitions and support from guidelines

Domain of care	Quality indicator	Support from ESC guidelines
	Main QI: The center should be part of a Network Organization with written protocols for rapid and efficient management covering the following points Single emergency phone number for the patient to be connected to a medical system for triage Pre‐hospital interpretation of ECG for diagnosis and decision for immediate transfer to a center with catheterization laboratory facilities, bypassing the Emergency Department Pre‐hospital activation of the catheterization laboratory Secondary QI (1): routine assessment of relevant times for the reperfusion process in STEMI patients (ie, times from “call to first medical contact,” “first medical contact to door,” “door to arterial access” and “door‐in door‐out” for centers without a catheterization laboratory on site) Secondary QI (2): the center should participate in a regular registry or program for quality assessment	Network: ESC GL, Class I, level B Written protocol: ESC STEMI GL Class I, level C Single phone number: no ESC GL to support this QI Pre‐hospital interpretation of ECG: ESC STEMI GL, Class I level B Pre‐hospital easy activation of the catheterization laboratory: ESC STEMI GL, level B Routine assessment of time to reperfusion for STEMI patients (time “call to first medical contact,” “first medical contact to door,” “door to device”): ESC STEMI GL, Class I, level C All hospital must record and monitor delay times: ESC STEMI GL, Class I, level B The center should participate regularly in a registry for quality assessment: ESC STEMI GL, Class I, level C Development of regional or national programs to measure performance indicators systematically and provide feedback to individuals hospitals: proposed as PM by ESC GL NSTE‐ACS 2015
Reperfusion‐invasive strategy	Main QI (STEMI 1): proportion of STEMI patients reperfused among eligible (onset of symptoms to diagnosis <12 hours) Main QI (STEMI 2): proportion of patients with timely reperfusion. Timely is defined as the following For patients treated with fibrinolysis: <30 minutes from diagnosis (FMC) to needle For patients treated with primary PCI and admitted to centers with catheterization laboratory facilities: <60 minutes from door to arterial access for reperfusion with PCI For transferred patients: door‐in door‐out time of <30 minutes Secondary QI (STEMI): the time between the diagnosis (FMC) and arterial access time (absolute value) for primary PCI Main QI (NSTEMI): proportion of patients with NSTEMI, and no contraindication, who receive coronary angiography within 72 hours after admission	Reperfusion STEMI patients—onset up to 12 hours: ESC STEMI GL, Class I, level A Timely reperfusion For patients treated with fibrinolysis: <60 minutes FMC to needle: ESC STEMI GL, Class I, level B For patients admitted to centers with catheterization laboratory facilities: <60 minutes door to balloon (passage of wire) for reperfusion with PCI: ESC STEMI GL, Class I, level B For patients transferred to a non PCI‐capable centre for primary PCI: ‐should bypass the emergency department: ESC STEMI GL, Class IIa, level B ‐<30 minutes door‐in door‐out: ESC revascularization GL, Class IIa, level B All hospitals must record and monitor delay times: ESC STEMI GL, Class I, level B Invasive strategy in moderate‐high risk patients: ESC NSTE‐ACS GL, Class I, level A
In‐hospital risk assessment	Main QI (1): proportion of patients with NSTEMI who have ischaemic risk assessment using the GRACE risk score. GRACE risk score should be assessed and the numerical value of the score recorded for all patients admitted with suspected NSTEMI Main QI (2): proportion of patients admitted with STEMI and NSTEMI who have bleeding risk assessment using the CRUSADE bleeding score. The CRUSADE bleeding score should be assessed and the numerical value of the score recorded for all patients admitted with STEMI and NSTEMI Main QI (3): proportion of patients with STEMI and NSTEMI who have assessment of left ventricular ejection fraction. Left ventricular ejection fraction should be assessed and the numerical value recorded for all patients admitted with STEMI and NSTEMI	The use of risk scores for estimating prognosis is recommended: ESC NSTE‐ACS GL, Class I, level A Use of the CRUSADE score … in patients undergoing coronary angiography: ESC NSTE‐ACS GL, Class IIb, level B Assessment of left ventricular ejection fraction: ESC STEMI GL, Class I, level B, ESC NSTE‐ACS GL, Class I, level B
Anti thrombotics during hospitalization	Main QI (1): proportion of patients with “adequate P2Y_12_ inhibition” defined as: number of patients discharged with prasugrel or ticagrelor or clopidogrel/patients eligible Eligible is defined as follows For ticagrelor: AMI patients without previous hemorrhagic stroke, high bleeding risk, fibrinolysis or oral anticoagulation For prasugrel: PCI‐treated AMI patients without previous hemorrhagic or ischemic stroke, high bleeding risk (patients ≥75 years or <60 kg body weight are also considered as high bleeding risk), fibrinolysis, or oral anticoagulation For clopidogrel: no indication for prasugrel or ticagrelor with no high bleeding risk Main QI (2): proportion of patients with NSTEMI treated with fondaparinux, unless candidates for immediate (≤2 hours) invasive strategy, or with eGFR ≥20 mL/min Secondary QI: proportion of patients with AMI discharged on dual antiplatelet therapy/patients with AMI without clear and documented contraindication	Ticagrelor in absence of contraindication for all patients regardless of initial strategy (ie, patients without previous hemorrhagic stroke, high bleeding risk, oral anticoagulation): ESC NSTE‐ACS GL, Class I, level B Prasugrel: in patients without previous hemorrhagic or ischemic stroke, high bleeding risk (patients ≥75 years, <60 kg body weight are also considered as high bleeding risk), oral anticoagulation, treated with PCI: ESC NSTE‐ACS GL, Class I, level B Clopidogrel: for patients who cannot receive ticagrelor or prasugrel or who require oral anticoagulation: ESC NSTE‐ACS GL, Class I, level B Fondaparinux is recommended as having the most favorable efficacy/safety profile regardless of the management strategy: ESC NSTE‐ACS GL, Class I, level B Irrespective of the revascularization strategy, a P2Y_12_ inhibitor is recommended in addition to aspirin for patients with AMI: ESC STEMI GL, Class I, level A, ESC NSTE‐ACS GL, Class I, level A
Secondary prevention‐discharge treatment	Main QI: proportion of patients with AMI discharged on statins, unless contraindicated, at high intensity (defined as atorvastatin ≥40 mg or rosuvastatin ≥20 mg) Secondary QI (1): proportion of patients with AMI and clinical evidence of heart failure or a LVEF ≤0.40 who are discharged on ACEI (or ARBs if intolerant of ACEI) unless contraindicated Secondary QI (2): proportion of patients with AMI and clinical evidence of heart failure or a LVEF ≤0.40 who are discharged on β‐blockers, unless contraindicated	Statins high intensity as early as possible, unless contraindication: ESC STEMI GL, Class I, level A, ESC NSTE‐ACS GL, Class I, level A β‐blocker therapy in patients with LVEF ≤0.40, unless contraindicated: ESC STEMI GL, Class I, level A, ESC NSTE‐ACS GL, Class I, level A ACE inhibitor in patients with LVEF ≤0.40 or heart failure, hypertension or diabetes: ESC STEMI GL, Class I, level A, ESC NSTE‐ACS GL, Class I, level A Use of aspirin, ticagrelor/prasugrel/clopidogrel, statins, β‐blocker and ACE inhibitor (in patients with LVEF ≤0.40 or heart failure), enrolment in cardiac rehabilitation at discharge: proposed as PM by ESC GL NSTE‐ACS 2015, no recommendation
Patient satisfaction	Main QI: feedback regarding the patient's experience is systematically collected for all patients. This should include the following points: Pain control Explanations provided by doctors and nurses (about the coronary disease, the benefit/risk of the discharge treatment, and medical follow‐up) Discharge information regarding what to do in case of a recurrence of symptoms and recommendation to attend a cardiac rehabilitation program (including smoking cession and diet counseling)	No ESC GL to support this QI Review paper from Anker et al. published in *Eur Heart J* in 2014 Participation in a well‐structured cardiac rehabilitation program: ESC NSTE‐ACS Gl, Class IIa, level A Smoking cessation advice/counseling: ESC STEMI GL, Class I, level C; proposed as PM by ESC GL NSTE‐ACS 2015, no recommendation Enrolment in a secondary prevention/cardiac rehabilitation program: proposed as PM by ESC NSTE‐ACS GL, 2015, no recommendation
Composite and outcome QI	Main QI (1): opportunity based CQI, with the following individual indicators The center is part of a network organization Proportion of patients reperfused among eligible (STEMI with FMC <12 hours after onset of pain) Coronary angiography in STEMI and NSTEMI patients at high ischemic risk and without contraindications Ischemic risk assessment using the GRACE risk score in NSTEMI patients Bleeding risk assessment using the CRUSADE risk score in STEMI and NSTEMI patients Assessment of LVEF before discharge Low dose aspirin (unless high bleeding risk or oral anticoagulation) Adequate P2Y_12_ inhibition (unless documented contraindication) ACEI (or ARB if intolerant of ACEI) in patients with clinical evidence of heart failure or an LVEF ≤0.40 β‐blockers (unless clear contraindication) in patients with clinical evidence of heart failure or an LVEF <0.40 High intensity statins Feedback regarding the patient's experience and quality of care is systematically collected for all patients Secondary CQI: all or the LVEF In patients without heart failure and with LVEF >0.40, CQI calculated on 3 individual QI Low dose aspirin P2Y_12_ inhibitor (unless documented contraindication) High intensity statins In patients with heart failure or with LVEF ≤0.40, CQI calculated on 5 individual QI Low dose aspirin P2Y_12_ inhibitor (unless documented contraindication) High intensity statins ACEI (or ARB if intolerant of ACEI) in patients with clinical evidence of heart failure or LVEF <0.40 β‐blockers (unless clear contraindication) in patients with clinical evidence of heart failure or an LVEF ≤0.40 Secondary outcome QI: 30‐day mortality, adjusted for the GRACE 2.0 risk score	No ESC GL to support this QI ESC NSTE‐ACS GL proposed “Performance measures”, but only individual indicators, no composite indicator

Abbreviations: ACEI, angiotensin‐converting enzyme inhibitor; AMI, acute myocardial infarction; ARB, angiotensin receptor blocker; CRUSADE, Can Rapid risk stratification of Unstable angina patients Suppress ADverse outcomes with Early implementation of the ACC/AHA guidelines; CQI, composite quality indicator; ECG, electrocardiogram; eGFR, estimated glomerular filtration rate; ESC, European Society of Cardiology; FMC, first medical contact; GL, guidelines; GRACE, Global Registry of Acute Coronary Events; LVEF, left ventricular ejection fraction; NSTE‐ACS, non‐ST‐segment elevation acute coronary syndrome; NSTEMI, non‐ST‐segment elevation myocardial infarction; PCI, percutaneous coronary intervention; PM, performance measure; QI, quality indicator; STEMI, ST‐segment elevation myocardial infarction.

*Source*: Adapted from Schiele et al. with permission from SAGE Ltd.^71^

Guidelines and hospital protocols should emphasize the importance of documenting patient history, which could affect NSTEMI diagnosis, and of regularly assessing the risks and benefits of therapies to suit the patient's clinical status, which may change over time.^54^ Furthermore, guidelines and protocols should include guidance on how to manage patients with dyspnea because some physicians are concerned about dyspnea related to ticagrelor use in their patients, given that dyspnea is more frequently reported in patients with ACS prescribed ticagrelor compared with clopidogrel.^73^, ^74^ The incidence of dyspnea in a real‐world setting has been shown to be greater than that reported in clinical trials and may lead to higher rates of ticagrelor discontinuation.^74^ However, given that there is evidence to suggest that dyspnea can resolve during inhibitor use, only in the case of persistent ticagrelor‐related dyspnea should drug discontinuation be considered.^73^

There is potential for reducing the impact of geographical variation on the availability of procedures, cardiologists, and medical treatment for patients with NSTEMI, and improving outcomes. In South Australia in 2001, the 30‐day mortality from MI was higher in rural areas than in urban areas (14% vs. 9%). By 2010, since the introduction of the regionalized Integrated Cardiovascular Clinical Network,^75^ incorporating cardiologist‐supported remote risk stratification and facilitated access to tertiary hospital‐based early invasive management, 30‐day mortality had improved to approximately 7% for both rural and urban areas.^76^

Physician education, and the introduction of guidelines and hospital protocols with quality assessment measures, could also help increase the use of guideline‐indicated treatments in regions and hospitals where suboptimal treatment of patients with NSTEMI is prevalent. However, suboptimal treatment as a result of the low number of catheterization laboratories, the high cost of medications, and the healthcare system infrastructure is more difficult to resolve, because it relates to the level of funding available. In the aforementioned study assessing the implications of the mode of patient presentation to hospitals, the authors suggested that the high‐risk patients presenting to community hospitals without the capability to carry out PCIs could be triaged at a more proximal time point to appropriate centers, to avoid unnecessary inter‐hospital transfers and ensure more timely cardiac catheterization.^67^ This could be a reasonable approach to improve treatment outcomes for patients with NSTEMI, which could be facilitated by existing regional platforms for the management of patients with STEMI.^67^

## 4 CONCLUSIONS

Patients with NSTEMI have a higher long‐term mortality risk than patients with STEMI but are often treated less aggressively, with those who have the highest ischemic risk often receiving the least aggressive treatment (the “treatment‐risk paradox”). The suboptimal treatment of patients with NSTEMI can be explained by the heterogeneity of patient characteristics, an underestimation of the high ischemic risk compared with bleeding risk by physicians, procedure availability, and policy. To address these challenges, potential approaches include: developing guidelines and protocols that include rigorous definitions of NSTEMI, risk assessment, and integrated quality assessment measures; providing education to physicians on the management of long‐term cardiovascular risk in patients with NSTEMI; and making stents and antiplatelet therapies more accessible to patients.

## DISCLOSURE OF INTEREST

P.A.: Research support and consultancy for AstraZeneca, Sanofi Aventis, Amgen, CSL, Boehringer Ingelheim, Bayer, Novartis, and Merck. I.A.: Speaking and consultancy for Amgen, AstraZeneca, Bayer, Daiichi Sankyo, Pfizer /Bristol‐Myers Squibb, Novartis, and Sanofi. O.A.: Speaking and consultancy for AstraZeneca, Bayer, Aspen, Boehringer‐Ingelheim, Pfizer, Abbot, Servier, Sanofi, Glaxo SKB, KRKA, Bristol‐Meyers Squibb, ACINO, The Medicines, Raipharm, Novartis, and Lilly. E.C.Z.: Research grants from AstraZeneca, Amgen, Bayer, Boehringer‐Ingelheim, Pfizer, Valentech, Novartis, Merck, Boston Scientific, Aspen, Biospifar, Servier, and Legrand. A.Y.Y.F.: Speaking and consultancy for AstraZeneca, Amgen, Bayer, Boehringer Ingelheim, Pfizer, Novartis, Roche Diagnostics, Siemens, Medtronic, Boston Scientific, and OrbusNeich Medical. Research grants from Boehringer Ingelheim, and Medtronic. K.F.A.‐H.: Speaking and consultancy for AstraZeneca, Sanofi, Amgen, Algorithim, and Roche. S.H.: Speaking for AstraZeneca, Bayer, Boehringer Ingelheim, Bristol Myers Squibb, Pfizer, Novartis, and Sanofi. M.A.B.S.K.A.K.: Speaking and consultancy for AstraZeneca, Boehringer Ingelheim, Bayer, Novartis, Servier, Abbott Vascular, Biosensors International, Aspen, and Merck Sharp & Dohme. R.S.‐R.: Speaking and consultancy for AstraZeneca. R.W.: Research grants and personal fees from AstraZeneca, Bayer, and Boehringer Ingelheim, and personal fees from Pfizer/Bristol Myers Squibb. H.Y.: Nothing to disclose.
